# Early mobilization in intensive care unit in Latin America: A survey based on clinical practice

**DOI:** 10.3389/fmed.2022.1005732

**Published:** 2022-11-21

**Authors:** Marisol Barros-Poblete, Saint-Clair Bernardes Neto, Vicente Benavides-Cordoba, Rodolfo P. Vieira, Manuel Baz, Joan-Daniel Martí, Martijn A. Spruit, Rodrigo Torres-Castro

**Affiliations:** ^1^Programa de Doctorado en Ciencias Médicas, Escuela de Graduados Facultad de Medicina, Universidad Austral de Chile, Valdivia, Chile; ^2^FACISA—Faculdade de Ciências de Saúde do Trairi, Federal University of Rio Grande do Norte, Natal, Brazil; ^3^Facultad de Ciencias de la Salud, Pontificia Universidad Javeriana Cali, Cali, Colombia; ^4^Evangelical University of Goias (Unievangélica), Goiás, Brazil; ^5^Brazil University, São Paulo, Brazil; ^6^Brazilian Institute of Teaching and Research in Pulmonary and Exercise Immunology (IBEPIPE), São Paulo, Brazil; ^7^Federal University of São Paulo (UNIFESP), São Paulo, Brazil; ^8^Área de Cuidados Intermedios, Departamento Clínico de Medicina, Facultad de Medicina, Hospital de Clínicas, Universidad de la República, Montevideo, Uruguay; ^9^Cardiovascular Surgery Intensive Care Unit, Hospital Clínic de Barcelona, Barcelona, Spain; ^10^Department of Research and Development, CIRO, Horn, Netherlands; ^11^Department of Respiratory Medicine, Faculty of Health, Medicine and Life Sciences, Maastricht University Medical Centre, NUTRIM School, Maastricht University, Maastricht, Netherlands; ^12^Departamento de Kinesiología, Facultad de Medicina, Universidad de Chile, Santiago, Chile; ^13^International Physiotherapy Research Network (PhysioEvidence), Barcelona, Spain

**Keywords:** early mobilization, intensive care unit, Latin America, survey, exercise

## Abstract

**Background:**

The application of early mobilization (EM) in intensive care units (ICUs) has shown to improve the physical and ventilatory status of critically ill patients, even after ICU stay. This study aimed to describe the practices regarding EM in ICUs in Latin America.

**Methods:**

We conducted an observational, cross-sectional study of professionals from all countries in Latin America. Over 3 months, professionals working in ICU units in Latin America were invited to answer the survey, which was designed by an expert committee and incorporated preliminary questions based on studies about EM recommendations.

**Results:**

As many as 174 health professionals from 17 countries completed the survey. The interventions carried out within each ICU were active mobilization (90.5%), passive mobilization (85.0%), manual and instrumental techniques for drainage of mucus secretion (81.8%), and positioning techniques (81%). The professionals who most participated in the rehabilitation process in ICUs were physiotherapists (98.7%), intensive care physicians (61.6%), nurses (56.1%), and respiratory therapists (43.8%). In only 36.1% of the ICUs, protocols were established to determine when a patient should begin EM. In 38.1% of the cases, the onset of EM was established by individual evaluation, and in 25.0% of the cases, it was the medical indication to start rehabilitation and EM.

**Conclusion:**

This report shows us that EM of critically ill patients is an established practice in our ICUs like in other developed countries.

## Introduction

Critical illness is a life-threatening multi-system process that can result in significant morbidity or mortality and a major global burden (1). More extended periods of immobilization increase the deleterious physiological effects of critical illness (2, 3). This may cause functional alterations, such as microvascular ischemia, catabolism, polyneuropathy with axonal degeneration, muscle weakness, lack of cardiovascular resistance, and dependence on basic activities of daily life (2) and syndromes like intensive care unit acquired weakness (ICUAW) defined as “clinically detected weakness in critically ill patients in whom there is no plausible etiology other than critical illness (4),” which, together with the complications of critical patients, can slow down ventilatory weaning, and lead to increased morbidity and mortality (5).

To improve short-term prognosis, intensive care should be delivered by an interdisciplinary team, including physicians, nurses, respiratory therapists, physical and occupational therapists, dieticians, social workers, spiritual care providers, and pharmacists (6, 7). Indeed, the application of early mobilization (EM) in intensive care units (ICUs) has shown to improve the physical and ventilatory status of critically ill patients (6, 8, 9), improving ICUAW, shortening their mechanical ventilation (MV) dependence, increasing ventilator-free days, and decreasing their ICU and hospital length of stay (10).

Thus, EM can reduce healthcare costs and morbidity while increasing health-related quality of life (11, 12). Moreover, growing evidence suggests that active mobilization and rehabilitation improve muscle strength and functional independence (13) and reduce delirium, especially if introduced within the first few days of ICU admission (14). Therefore, EM involves timely progression during critical illness through a series of activities from active range of motion to full ambulation (3, 7), and is defined as the application of physical activity within the first 2–5 days of critical illness or injury (15), being optimal to improve the results by starting between 48 and 72 h after the start of MV if possible (16).

Based on this, there are different European experiences (16, 17), and, as such, EM has been recommended by scientific societies around the world (17, 18). There is also experience in low-income countries, as reported by Shpata, which may be more similar to what happens in Latin America, where health professionals deal with challenges such as poverty, low salaries, and low quality of employment (19, 20). Additionally, health systems vary across Latin American countries, and a mix of public and private ICUs exist, sometimes with uneven resource distribution (21). Thus, the availability of physiotherapy staff in the ICU may significantly vary among units and hospitals and, importantly, differ across Latin countries. The heterogeneity of the countries in Latin America makes us hypothesize that EM is implemented to different degrees depending on the country. Therefore, this study aimed to describe and compare the practices regarding EM in ICUs from different countries and regions in Latin America.

## Materials and methods

### Study design

We conducted an observational, cross-sectional study of professionals from all countries in Latin America. From November 2019 to January 2020, intensive care physicians, physiotherapists, nurses, and other professionals working in ICUs in Latin America were invited to answer a dedicated survey. Participants were invited using Asociación Latinoamericana de Tórax (ALAT) registry and by the society’s social network (Facebook, Instagram, and Twitter) and personal contacts. This study was designed and coordinated by the Respiratory Care Department of the ALAT and approved by the ALAT institutional board (Act. 280818, meeting in Mexico City, Mexico). We excluded all responses from professionals who did not work in ICUs and who did not belong to Latin America. In the event that two or more people answered in the same center, only the first answer was considered.

### Survey

The survey was designed by an expert committee that incorporated preliminary questions based on studies about EM recommendations (17, 18). After a draft revision of the Respiratory Care department, it was sent to five independent reviewers selected for their experience in the area; after receiving their recommendations, some questions were reformulated, added, or deleted, and the final version was obtained. After piloting the survey in three centers, the final version included 13 questions under the following domains: demographics, clinical practice in each unit, patient selection criteria for EM and outcomes, and subsequent follow-up (Supplementary File 1). In addition, we followed the checklist for reporting results of internet e-survey (CHERRIES) recommendations for preparing online surveys (22).

The survey was distributed in Spanish and Portuguese, and, according to the responses obtained, the results were filtered by services and centers; only the first answer per service was considered. The survey was designed with an open survey system. The participants’ responses were stored with a number on an encrypted server.

### Statistical analysis

Descriptive statistics were calculated and reported as frequencies and proportions for categorical variables and means and standard deviations for continuous normally distributed variables. Statistical differences between countries were calculated using the Kruskal–Wallis, Fisher’s exact, and Mann–Whitney tests. Statistical significance was defined as a two-sided *P* < 0.05. Statistical analyses were completed using Graph Pad Prism 9 for macOS statistical software version 9.0 (GraphPad Software, La Jolla, CA, USA).

## Results

### Demographics

**FIGURE 1:**
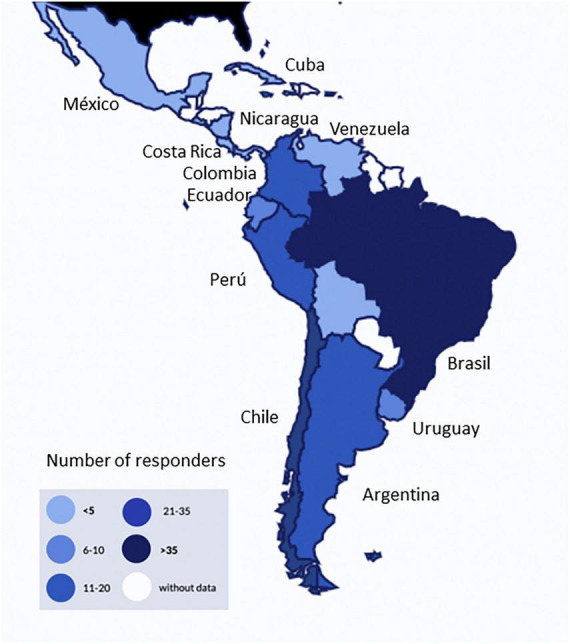
Distribution of responses by country.

We obtained 174 surveys from health professionals in 17 different countries. Three responses were excluded due to non-Latin American sources, and, from the 171, we finally selected 155 for analysis after applying exclusion criteria. The countries which the greatest data participations were Brazil, Chile, Colombia, and Argentina (Figure 1). The main professionals who responded to the survey were: physiotherapists (80%), respiratory therapists (8%), intensive care physicians (8%), and nurses (4%).

### Evaluation and interventions

The main interventions carried out within each unit were active mobilization (90.5%), followed by passive mobilization (85.0%), manual and instrumental techniques for drainage of mucus secretion (81.8%), and positioning techniques (81%) (see Table 1).

**TABLE 1:** Interventions performed in the intensive care unit (Question 1) (*n* = 155, expressed in percentage).

Interventions	*N* (%)
Active mobilization	140 (90.5)
Passive mobilization	132 (85.2)
Manual or instrumental techniques of mucus secretion drainage	127 (81.8)
Training of respiratory muscles	114 (73.8)
Neuromuscular electrical stimulation	30 (19.6)
Passive/mechanically assisted mobilization	36 (23.7)
Functional activities	101 (65.1)
Active mobilization mechanical resisted	101 (65.1)
Manual resisted mobilization	107 (69.6)
Upper limb muscle strength training	83 (53.5)

Regarding the functional activities that were carried out in the ICU and that included changes of position, those that were carried out were: rotating the supine position in bed, seating on a chair, seating at the edge of the bed, standing with manual assistance, standing assisted with “tilt table” static walking and walking inside the unit. In general, the interventions that were performed the least of these were: standing the patient with manual assistance and performing it with the assistance of a tilt table (58.0 and 27.0%, respectively).

In this point, we found differences between Latin American regions; particularly, standing with manual assistance was carried out in the Southern Cone regions (Chile, Argentina, and Uruguay), and it is practically not performed at all in Brazil (83.6 vs. 2.6%, *p* < 0.0001), but in the Southern Cone region they did perform assisted standing with tilt table, without differences between Brazil and the other countries (28.9% vs. 34.5%, *p* = 0.65). The type of patients where position changes included verticalization were those who had a safer airway, such as tracheostomised (TCT) patients, whether they were or were not on MV vs. patients with orotracheal tube (OTT) regardless of being on MV or not (73.5 and 85.9% vs. 34.1 and 24.5%, *p* < 0.001). The patients under non-invasive ventilation (NIV) performed verticalization in a proportion comparable to patients with TCT with MV (74.8% vs. 73.5%, *p* = 0.398) but higher than OTT with ventilation (74.8% vs. 34.1%, *p* < 0.0001). Compared to the whole cohort of patients, those needing dialyses were the least likely to be provided verticalization in the ICU (12.9%). When we analyzed regional differences, we observed that the units of the Southern Cone mobilized patients with OTT more than the Brazil units (*p* < 0.0001).

### Rehabilitation teams

**FIGURE 2:**
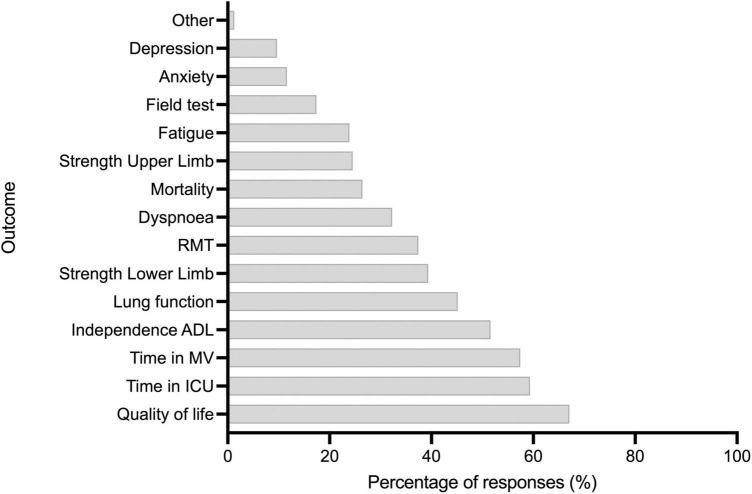
Question 11: Which of the following results do you consider to be the most important after rehabilitation. RMT, Respiratory muscle training; ADL, Activities of daily living; MV, Mechanical ventilation; ICU, Intensive care unit.

Regarding the professionals who participated in the rehabilitation process in ICUs, in 98.7% of the responders, the physiotherapist was part of the team, followed by the intensive care physician (61.6%), nurses (56.1%), and respiratory therapists (43.8%). On the other hand, the speech and language therapists participated in 21.9% of cases, and the occupational therapist participated in only 18.1% of the cases. Other medical specialists like cardiologists, pulmonologists, and physiatrists participated in less than 25%, and other professionals, such as nutritionists, pharmaceutical chemicals, psychologists, and social workers, participated in less than 15% of the cases (Figure 2).

### Practices

By carrying out mobilization and rehabilitation therapy, over two-third of the physiotherapist respondents (71.6%) provided rehabilitation and EM activities in conjunction with secretion drainage or ventilatory therapy activities all together; that is, they performed comprehensive attention, while only 27.0% provided them separately. They were in charge of a mean of 15.3 ± 10.9 beds (with a range of between 3 and 75 beds). There was no difference between Brazil and the Southern Cone (16.9 ± 12.7 vs. 16.0 ± 9.8, *p* = 0.35). Concerning the time spent developing rehabilitation activities, 45.1% of the responders had indefinite time to attend to their patients, 36.7% had less than 30 min, and 18.2% had more than 30 min. When we compared those having indefinite time to develop rehabilitation activities, the minority of professionals had an indeterminate time to do them (*p* = 0.034), and, once again, there were no differences between Brazil and the countries of the Southern Cone.

We found protocols established in the unit to determine when a patient should begin EM in 36.1%. In 38.1% of the cases, the classification was performed on a patient-by-patient basis through a general evaluation of when to start EM, while 25.0% of the cases presented the medical indication to start rehabilitation and EM. While in the Southern Cone, it was established mainly by evaluation of the patient on a case-by-case basis, in 47.2% of the cases, only 20% of the professionals had a protocol to establish when to start the EM, which occurred on the contrary among 92.1% of the Brazilian professionals (*p* < 0.0001).

As many as 97% (151) of the responders considered hemodynamic stability as a necessary element to be able to start EM, 78% (121) considered respiratory stability, 61% (94) considered neurologic stability, and 50% (77) the need to start EM of tests in the normal range; we do not find differences between regions.

The patients’ selection included a broad spectrum of pathologies, like oncological diseases, post-neurological surgery, post-vascular surgery, post-thoracic surgery, post-abdominal or thoracic surgery patients, and patients with metabolic, neurological, and respiratory diseases. In addition, 51.6% of the responders have systems or units to continue rehabilitation after discharge from the ICU, vs. 48.4% who do not (*p* = 0.28).

### Parameters to establish results after rehabilitation

**FIGURE 3:**
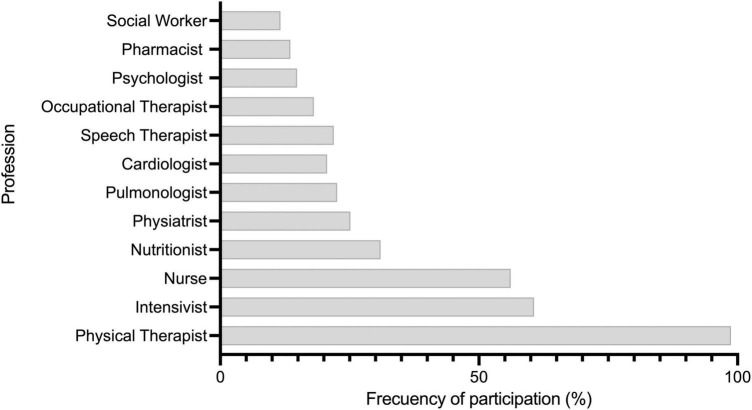
Question 2: Which professionals participate in the rehabilitation intervention plan for patients in intensive care units?

Finally, the most relevant parameters when it comes to establishing results after mobilization were quality of life (67.0%), time in the ICU (59.3%), time in MV (57.4%), and level of independence (51.6%). By contrast, the least relevant was the outcome in field tests, such as the 6-min walking test (17.4%), index of anxiety (11.6%), and index of depression (9.6%) (Figure 3).

## Discussion

This report is the first Latin American experience that involved health professionals from different countries. According to previous reports, in Latin America, there exist around 494 ICUs, but this includes 300 which are in Central America, and not included in our study (23). If we consider this in the rest of the countries (Chile, Argentina, Brazil, Peru, Paraguay, Uruguay, Colombia, and Ecuador), it will be approximately 194 ICUs. Our results may represent 79% of this, but this could be overestimated.

In this study, the professionals involved in the rehabilitation process were mainly physiotherapists and nurses, followed by intensive care physicians. Only a few places included speech or occupational therapists, which is similar to the reality of other countries. Our results are similar to high-income developed countries, such as the German and Swiss experiences that describe the same professionals primarily involved (24, 25). However, in more than 90% of the rehabilitation teams, the presence of the physiotherapist is encouraging, compared to realities in other countries, similar in development (20).

Trained health professionals are essential members of the interprofessional ICU team who can assess and manage intubated and spontaneously breathing patients, between them, namely intensive care physicians, nurses, and physiotherapists (7, 26). Traditionally, physiotherapy management’s mainstay was focused on preventing and managing respiratory complications such as sputum retention, atelectasis, and facilitation of ventilatory weaning and/or prevention of reintubation (27). However, a growing body of evidence also suggests physiotherapy at the early stages of the acute disease as a critical factor in preventing and counterbalancing ICUAW (9, 14, 26), and has been included in recommendations from evidence-based interprofessional team management strategies such as the ABCDEF bundle with “E” from EM (28). In addition, measures are being incorporated in Latin America to see the high percentage of participation of physical therapists.

Undoubtedly, the common finding of our study was the heterogeneity of EM in our region. An important point of concern is that most of the responses show us that not all professions are present in multidisciplinary teams, with a prevalence close to 20% of speech and language therapists and occupational therapists. These two professionals are essential in teams that have patients with tracheostomy or delirium, who are usually present in ICUs (6). On the other hand, the participation of medical specialties linked to rehabilitation such as physiatrist or pneumology was very low (less than 25%). It is necessary to move toward multidisciplinarity to obtain the maximum benefit for patients (6, 7).

Patients with a safe airway can perform functional activities and activities involving verticalization significantly more if they are with TCT or NIV than with OTT; this can be explained in part by the more frequent use of deep sedation in these patients with greater physiological instability or shorter stay in the unit (24) or it is possible that having a safer airway with NIV and TCT than a TTO that can move easily, and also limits the possibilities of mobilization and verticalization. In general, mobilization activities used by professionals in Latin America are similar to other countries (24), highlighting the little use of passive or mechanically assisted mobilization and neuromuscular stimulation, although the latter has been reported as effective and safe (24), this likely due to lack of technological resources, of theoretical, practical training as well as insufficient personnel.

Also, this can be seen reflected in the number of beds per professional, which, in this experience, is similar to Turkish (15 ± 20.7) (29) greater than Swiss and German reality (6.6 ± 3.6 and 12.7 ± 8.3) but is less than the United States having 23 ± 7 beds per professional (25, 30, 31).

On the other hand, we observed that most of the units do not have a protocol for entering EM programs, which in general is lower than that reported by other authors (24, 25), except for the area of Brazil, wherein 90% of cases have a protocol to determine the beginning of EM. As a result, the physiotherapist depends on the medical indication or the individual evaluation of each patient, which can delay EM, which may contribute to not meeting the recommendation to start EM 48–72 h post-MV start for improving clinical outcomes (24). Studies that implemented EM protocols have seen reduced ICU and hospital length and decreased rates of delirium (9).

When evaluating the professionals’ criteria to determine favorable conditions to start EM, a large number of the professionals considered hemodynamic stability the most relevant, which is considered the main barrier for starting EM and consistent with that reported by other authors (24), followed by the neurological stability related to the level of collaboration (32). This difficulty in starting with EM has also been reported in the literature (3, 24, 25), for example, excessive sedation, lack of personnel, concern for safety, insufficient guidelines and protocols, limited equipment, and inadequate staff training (33). Although the cost-effectiveness of EM is well-supported, it has not been considered a priority due to a large number of staff and the evidence that absorbs their profits (34). These difficulties can be optimized by working as a team, which we see as effective according to the responses of our surveys in Latin America.

Finally, the main parameters for establishing outcomes after the rehabilitation included time in the ICU and time in MV supporting evidence, where it has been pointed out that rehabilitation interventions reduced the ICU stay and also the duration of MV (24). However, these interventions were not directly related to an improvement in the long-term quality of life, as the latter is one of the frequently measured parameters in our units, and its use should be reviewed. In addition to strengthening interdisciplinary work with intensive medicine, nursing, and nutrition, the results obtained in general terms will depend on the articulation of all.

## Strengths and limitations

The main strength of our study is that it allows us to have a global picture of what happens with EM in Latin America, where there are practically no data. This information is useful for scientific societies to establish a starting point for recommendations and clinical guidelines.

On the other hand, our study had several potential limitations; first, we applied this survey before the COVID-19 pandemic, a situation that modified the reality of the ICU, and increased the staff’s workload and the reality of the patients. Second, Central American countries are under-represented with low participation in the survey. Third, we do not know precisely the total number of ICUs in Latin America, so we cannot know with precision the representativeness of our results. Fourth, our survey collected data on the execution of each strategy, but not on the number of times it is carried out each day. Undoubtedly, early mobilization is similar to other interventions that depend on the dose to achieve the desired effect. Finally, we included different types of ICU and rehabilitation strategies could vary depending on the type of patient (e.g., neurological, respiratory, oncological, etc.), the type of ventilatory support (e.g., invasive or non-invasive), and the type of other pharmacological or medical treatments (e.g., vasoactive amine and dialysis utilization).

## Conclusion

This report from Latin America shows us that the physical rehabilitation, especially EM, of critically ill patients is an established practice that is present in our ICU like in other developed countries, demonstrating the strengths of the system such as the incorporation of health professional experts on EM and the use of various techniques including functional activities and position changes. Finally, it points out the areas that we have to continue working on in the future: developing protocols for EM, follow-up systems after leaving the ICU, staff training, and incorporation of rehabilitation professionals into the early rehabilitation and patient care team at ICU to allow the most comprehensive rehabilitation as possible.

## Data availability statement

The raw data supporting the conclusions of this article will be made available by the authors, without undue reservation.

## Ethics statement

This study was approved by the ALAT Institutional Board (Act. 280818, meeting in Mexico City, Mexico). The patients/participants provided their written informed consent to participate in this study.

## Author contributions

MB-P: conceptualization, formal analysis, investigation, writing—original draft, and writing—review and editing. S-CB, VB-C, RV, and MB: investigation, writing—original draft, and writing—review and editing. J-DM: writing—original draft and writing—review and editing. MS: methodology, writing—original draft, and writing—review and editing. RT-C: conceptualization, methodology, formal analysis, writing—original draft, and writing—review and editing. All authors contributed to the article and approved the submitted version.
