# Supplementary material for: Early mobilization in intensive care unit in Latin America: A survey based on clinical practice
Source: Front Med (Lausanne). 2022 Nov 21;9:1005732. doi: 10.3389/fmed.2022.1005732 (PMC9720404; doi:10.3389/fmed.2022.1005732)
Supplement: Supplementary file 1 [file Table_1.DOCX]

**Supplementary material 1. SURVEY OF PHYSICAL REHABILITATION IN CRITICAL PATIENTS IN LATIN AMERICA**

Dear Professional,

Early mobilization is recognized as an important part of the care of patients in intensive care units. The respiratory care department of the Latin American Association of Thorax (ALAT) wishes to carry out a survey of the reality of early mobilization in critically ill patients in Latin America. That's why we want to invite you to answer a small questionnaire about your experience in this area. This will provide us important information on the structure, content and organization of early mobilization to the development of this area.

Sincerely,

Department of Respiratory Care, Latin American Thorax Association

Department of Intensive Care, Latin American Thorax Association

*1. In your intensive care unit, which of the following interventions are performed? (multiple answer are possible)*

Manual and instrumental techniques for mucus secretion drainage

Positioning

Active mobilization

Passive mobilization

Training of respiratory muscles

Neuromuscular electrical stimulation

Passive or mechanically assisted mobilization (For example MotoMed)

 Functional activities (for example transfers, transfers, etc.)

Active mobilization mechanically resisted (For example elastic band or bicycle)

Resisted mobilization with manual resistance by the professional

.  Upper-limb muscle strength training (elastic bands, dumbbells, etc.

 Other: ____________________

*2. Which professionals participate in the rehabilitation plan of patients in the ICU? (multiple answers are possible)*

Medical specialist

 Intensivist

 Pulmonologist

 Physiatrist / Rehabilitator

 Cardiologist

 Other:________________

Physiotherapist / Kinesiologist / Physical Therapist / Respiratory Therapist

Nurse

Occupational therapist

Social worker

Psychologist

 Dietitian / Nutritionist

Speech pathologist / Speech therapist

Pharmaceutical chemist

Other: ____________________

*3. Early mobilization interventions and physical rehabilitation of patients in ICU are performed:*

In conjunction with the drainage secretions activities and/or respiratory therapy

Separately from the drainage secretions activities and/or respiratory therapy

*4. How many beds are in the service that you work?*  ***_______***

*5. On average, how much time do you have to work in early mobilization per patient in a normal workday, without regard the administrative work ?*

0 a 10 minutes

11 a 15 minutes

16 a 20 minutes

21 a 30 minutes

31 a 40 minutes

41 a 50 minutes

51 a 60 minutes

Undetermined according to the patient's requirements.

*6. To select the patients and initiate the early mobilization in ICU*:

You have a protocol established by the unit and/or published in the literature

You don´t have an established protocol, each patient is evaluated by the team and decide if the patients are eligible for early mobilization.

In your unit, you start the early mobilization once you have the primary medical indication to start evaluating and mobilize the patient.

*7. About early mobilization, what criteria do you use to determine stabiliby and favorable condition for early mobilization? (multiple answer are possible):*

Hemodynamic stability.

Respiratory stability.

Neurological stability.

Appropriate exams.

*8. What kind of patients do not apply for the rehabilitation program in your ICU? (Please check all applicable options).*

Respiratory diseases

Cardiovascular diseases

Neurological diseases

Metabolic diseases

Orthopedic Post-surgical patients

Neurological Post-surgical patients

Abdominal Post-surgical patients

Vascular Post-surgical patients

Thoracic Post-surgical patients

Cardiac Post-surgical patients

Oncological diseases

Others: ______________________

*9. Regarding the positioning and changes of position mark those that your performance in your unit*:
 In bed (supine, decubitus, turns)

 sit in an armchair

sit on the edge of the bed

stand up by the patients

stand up assisted with a tilt table

March stationary

walk around the room or through the unit

*10. Select those patients in whom you make changes of position that involve verticalization ( sit on the edge of bed and standing):*

Patients with non-invasive mechanical ventilation

Tracheostomized patients with ventilation

Tracheostomized patients without mechanical ventilation

 Patients with TOT with mechanical ventilation

Patients with TOT without ventilation

 Patients during dialysis

Others: ________________________________

*11. To evaluate the patient's response to the early rehabilitation program which of the following outcomes do you consider the most important (mark 5 please):*

 Quality of life

Dyspnoea

Fatigue

Depression

Anxiety

Field test (TM6, SWT, ESWT)

Strength of lower extremities

 Strength of upper extremities

Pulmonary function

Resistance on the bicycle

Strength of inspiratory muscles

 Functionality in activities of daily life

Time in mechanical ventilation

Time of stay in ICU

Mortalilty

 Other: ___________________

*12. Do you have in your hospital o unit systems to continue the rehabilitation program after discharge for the ICU?*

*No*

*Yes . Wich?:__________________________*

**Spanish version**

**ENCUESTA DE REHBITALICIÓN FÍSICA EN PACIENTES CRITICOS EN LATINOAMERICA**

Estimado profesional,

La movilización temprana se reconoce como una parte importante de la atención de pacientes en unidades de cuidados intensivos. El departamento de atención respiratoria de la Asociación Latinoamericana de Tórax (ALAT) desea realizar una encuesta sobre la realidad de la movilización temprana en pacientes críticos en América Latina. Es por eso que queremos invitarlo a responder un pequeño cuestionario sobre su experiencia en esta área. Esto nos proporcionará información importante sobre la estructura, el contenido y la organización de la movilización temprana para el desarrollo de esta área.

Sinceramente,

Departamento de Atención Respiratoria, Asociación Latinoamericana del Tórax

Departamento de Cuidados Intensivos, Asociación Latinoamericana de Tórax

*1. En la unidad de cuidados intensivos que ud trabaja se realiza alguna de las siguientes intervenciones kinésicas? se permiten múltiples respuestas.*

Técnicas manuales e instrumentales para el drenaje de secreción de moco

Posicionamiento

Movilización activa

Movilización pasiva

Entrenamiento de los músculos respiratorios

Estimulación eléctrica neuromuscular

Movilización pasiva o asistida mecánicamente (por ejemplo, MotoMed)

Actividades funcionales (por ejemplo, transferencias, transferencias, etc.)

Movilización activa resistida mecánicamente (por ejemplo, banda elástica o bicicleta)

Movilización resistida con resistencia manual por el profesional

Entrenamiento de fuerza muscular de las extremidades superiores (bandas elásticas, pesas, etc.)

Otro: ____________________

*2. Qué profesionales participan dentro del plan de intervención de rehabilitación de los pacientes en UCI? Se permiten múltiples respuestas.*

Médico especialista

Intensivista

Broncopulmonar

Cardiologo

  Fisiatra

De outros:________________

(broncopulmonar/fisiatra/ cardiologo/psiquiatra)

Fisioterapeuta/Kinesiólogo/Terapeuta Físico

Terapeuta ocupacional

Trabajador social

Psicólogo

Dietista/Nutricionista

Fonoaudiologo

Farmacéutico

Enfermera(o)

Otro: ____________________

*3.Las intervenciones de movilización precoz y rehabilitación física de los pacientes en UCI Ud. las realiza:*

En conjunto con las actividades de drenaje de secreciones y/o terapia ventilatoria

En forma separada de las actividades de drenaje de secreciones y/o terapia ventilatoria

*4. Cuántas camas tiene el servicio que Ud. trabaja? ______*

5. En promedio ¿Con *cuánto tiempo cuenta Ud. para trabajar en movilización precoz con cada paciente, sin considerar el tiempo utilizado en labores administrativa?.*

0 a 10 minutos

11 a 15 minutos

 16 a 20 minutos

 21 a 30 minutos

 31 a 40 minutos

41 a 50 minutos

 51 a 60 minutos

Indeterminado según los requisitos del paciente

*6. Para seleccionar a los pacientes e iniciar la movilización precoz de un paciente en UCI Ud:*

Cuenta con un protocolo establecido por la unidad y/o publicado por la literatura

Ud no cuenta con un protocolo cada paciente se evalúa para determinar si el paciente puede comenzar con movilización precoz

En su unidad, la movilización precoz se inicia una vez que esté la indicación médica primaria para comenzar a evaluar y tanto a movilizar al paciente.

*7. Respecto a la movilización precoz, que criterios usa Ud. para determinar la estabilidad y y las condiciones faorales para realizar movilización precoz (se permiten multiples respuestas.*

*Estabilidad hemodinámica*

*Estabilidad respiratoria*

*Estabilidad neurológica*

*Exámenes dentro de rangos normales.*

*8. ¿Qué tipo de pacientes no son incluídos en su programa de rehabilitación en UCI? (Por*

*favor marque todas las opciones aplicables)*

*Enfermedades respiratorias*

*Enfermedades cardiovasculares*

*Enfermedades neurologicas*

*Enfermedades metabolicas*

*Pacientes post cirugía traumatologica*

*Pacientes post cirugía neurologica*

*Pacientes post cirugía abdominal*

*Pacientes post cirugía vascular*

*Pacientes post cirugía toracica*

*Pacientes post cirugía cardiaca*

*Enfermedades oncologicas*

*Otras:__________________________*

*9. Respecto al posicionamiento y los cambios de posición marque aquellas que realiza Ud. en su unidad:*

En cama, supino, decúbitos y giros.

Sedente en sillón

Paso a sedente borde cama

Bípedo por el paciente

Bípedo asistido con tilt table

Marcha estática

Marcha por la unidad.

*10. Seleccione aquellos pacientes en que Ud. realiza los cambios de posición que implican verticalización (Sedente al borde cama y bipedestación):*

Pacientes con ventilación mecánica no invasiva

Pacientes traqueostomizados con ventilación mecánica

Pacientes traqueostomizados sin ventilación mecánica

Pacientes con tubo orotraqueal con ventilación mecánica

Pacientes con tubo orotraqueal sin ventilación mecánica

Pacientes durante diálisis

Otros: _______________________________________

*11. Para evaluar la respuesta a la rehabilitación temprana ¿Cuál de los siguientes resultados usted consideras es la más importante (marcar* ***5*** *por favor)*

Calidad de Vida

Disnea

Fatiga

Depresión

Ansiedad

Test de campo (TM6, SWT, ESWT)

Fuerza de extremidades inferiores

Fuerza de extremidades superiores

Función pulmonar

Resistencia en la bicicleta

Fuerza de los músculos inspiratorios

Independencia en actividades de la vida diaria

Tiempo en ventilación mecánica

Tiempo de estadía de UCI

Mortalidad

Otro: ___________________

12. Tiene en su hospital o unidad sistemas para continuar con la rehabilitación posterior al alta de la UCI.

No

Si ¿Cuál?

**PORTUGUESE VERSION**

INQUÉRITO DE REABILITAÇÃO FÍSICA EM PACIENTES CRÍTICOS NA AMÉRICA LATINA

Caro Profissional,

A mobilização precoce é reconhecida como parte importante do cuidado dos pacientes em unidades de terapia intensiva. O departamento de cuidados respiratórios da Associação Latino-Americana de Tórax (ALAT) deseja realizar um levantamento da realidade da mobilização precoce em pacientes críticos na América Latina. É por isso que queremos convidá-lo a responder um pequeno questionário sobre sua experiência nessa área. Isso nos fornecerá informações importantes sobre a estrutura, o conteúdo e a organização da mobilização precoce para o desenvolvimento dessa área.

Atenciosamente,

Departamento de Cuidados Respiratórios da Associação Latino-Americana de Tórax

Departamento de Terapia Intensiva da Associação Latino-Americana de Tórax

1. Na sua unidade de terapia intensiva, quais das seguintes intervenções são realizadas? (várias respostas são possíveis)

 Técnicas manuais e instrumentais de drenagem de secreções de muco

Posicionamento

Mobilização ativa

Mobilização passiva

Treinamento de músculos respiratórios

Estimulação elétrica neuromuscular

Mobilização passiva ou mecanicamente assistida (Por exemplo, MotoMed)

Atividades funcionais (por exemplo transferências, transferências, etc.)

Mobilização ativa resistida mecanicamente (Por exemplo, elástico ou bicicleta)

Mobilização resistida com resistência manual pelo profissional

Treinamento de força muscular nos membros superiores (elásticos, halteres, etc.)

Outros: ____________________

2. Quais profissionais participam do plano de reabilitação de pacientes na UTI? (várias respostas são possíveis)

  Médico especialista

Intensivista

Pneumologista

Fisiatra / Reabilitador

Cardiologista

De outros:________________

 Fisioterapeuta / Fisioterapeuta / Fisioterapeuta / Terapeuta Respiratório

 Enfermeira

Terapeuta ocupacional

Psicólogo

Assistente social

Nutricionista / Nutricionista

Fonoaudióloga / Fonoaudióloga

Químico farmacêutico

De outros: ____________________

3. Intervenções precoces de mobilização e reabilitação física de pacientes em UTI são realizadas:

 Em conjunto com as atividades de secreções de drenagem e / ou terapia respiratória

 Separadamente das atividades de secreções de drenagem e / ou terapia respiratória

4. Quantas camas estão no serviço que você trabalha? ______

5. Em média, quanto tempo você tem que trabalhar na mobilização precoce por paciente em um dia de trabalho normal, sem considerar o trabalho administrativo?

  0 a 10 minutos

11 a 15 minutos

16 a 20 minutos

 21 a 30 minutos

31 a 40 minutos

 41 a 50 minutos

51 a 60 minutos

 Indeterminado de acordo com os requisitos do paciente.

6. Para selecionar os pacientes e iniciar a mobilização precoce na UTI:

 Você tem um protocolo estabelecido pela unidade e / ou publicado na literatura

Você não tem um protocolo estabelecido, cada paciente é avaliado pela equipe e decide se os pacientes são elegíveis para mobilização precoce.

 Na sua unidade, você inicia a mobilização precoce assim que tiver a indicação médica primária para começar a avaliar e mobilizar o paciente.

7. Sobre a mobilização precoce, que critérios você usa para determinar a condição estável e favorável para a mobilização precoce? (múltiplas respostas são possíveis):

 Estabilidade hemodinâmica

 Estabilidade Respiratória

 Estabilidade neurológica.

 Exames apropriados.

8. Que tipo de pacientes não se inscreve no programa de reabilitação em sua UTI? (Por favor, verifique todas as opções aplicáveis).

 Doenças respiratórias

 Doenças cardiovasculares

 Doenças neurológicas

 Doenças metabólicas

Pacientes pós-cirúrgicos ortopédicos

Pacientes pós-cirúrgicos neurológicos

Pacientes pós-cirúrgicos abdominais

 Pacientes pós-cirúrgicos vasculares

Pacientes pós-cirúrgicos torácicos

 Pacientes pós-cirúrgicos cardíacos

 Doenças oncológicas

 Outras: ______________________

9. Em relação ao posicionamento e mudanças de posição marque aqueles que o seu desempenho em sua unidade:

Na cama (supino, decúbito, voltas)

sentar em uma poltrona

sentar na beira da cama

de pé pelos pacientes levantar-se assistido com uma mesa de inclinação

marcha estacionária

março ao redor da sala ou através da unidade

10. Selecione os pacientes nos quais você faz mudanças de posição que envolvem a verticalização (sentar na beira da cama e em pé):

 Pacientes com ventilação mecânica não invasiva

 Pacientes traqueostomizados com ventilação

Pacientes traqueostomizados sem ventilação

Pacientes com TOT com ventilação

 Pacientes com TOT sem ventilação

Pacientes durante diálise

 Outras: ________________________________

11. Para avaliar a resposta do paciente ao programa de reabilitação precoce, qual dos seguintes resultados você considera o mais importante (marca 5, por favor):

Qualidade de vida

 Dispneia

Fadiga

 Depressão

 Ansiedade

Teste de campo (TM6, SWT, ESWT)

 Força das extremidades inferiores

Força das extremidades superiores

Função pulmonar

 Resistência na bicicleta

Força dos músculos inspiratórios

Funcionalidade nas atividades da vida cotidiana

Tempo na Ventilacion mecanica

 Tempo de permanência na UTI

 Mortalilty

De outros: ___________________

12. Você tem em seu hospital o sistema de unidades para continuar o programa de reabilitação após a alta da UTI?

 Não

 Sim . Qual?: __________________________
